# Exo-Metabolites of *Phaseolus vulgaris*-Nodulating Rhizobial Strains

**DOI:** 10.3390/metabo9060105

**Published:** 2019-05-30

**Authors:** Diana Montes-Grajales, Nuria Esturau-Escofet, Baldomero Esquivel, Esperanza Martinez-Romero

**Affiliations:** 1 Centro de Ciencias Genómicas, Universidad Nacional Autónoma de México, Cuernavaca 62210, Mexico; 2 Instituto de Química, Universidad Nacional Autónoma de México, Mexico City 04510, Mexico; besquivel@iquimica.unam.mx; 3 Environmental and Computational Chemistry Group, University of Cartagena, Cartagena 130015, Colombia; dmontesg@unicartagena.edu.co

**Keywords:** rhizobia, nitrogen fixation, nuclear magnetic resonance, exo-metabolomics

## Abstract

Rhizobia are able to convert dinitrogen into biologically available forms of nitrogen through their symbiotic association with leguminous plants. This results in plant growth promotion, and also in conferring host resistance to different types of stress. These bacteria can interact with other organisms and survive in a wide range of environments, such as soil, rhizosphere, and inside roots. As most of these processes are molecularly mediated, the aim of this research was to identify and quantify the exo-metabolites produced by *Rhizobium etli* CFN42, *Rhizobium leucaenae* CFN299, *Rhizobium tropici* CIAT899, *Rhizobium phaseoli* Ch24-10, and *Sinorhizobium americanum* CFNEI156, by nuclear magnetic resonance (NMR). Bacteria were grown in free-living cultures using minimal medium containing sucrose and glutamate. Interestingly, we found that even when these bacteria belong to the same family (*Rhizobiaceae*) and all form nitrogen-fixing nodules on *Phaseolus vulgaris* roots, they exhibited different patterns and concentrations of chemical species produced by them.

## 1. Introduction

Exo-metabolomics may contribute to the understanding of the bacteria communication mechanisms through the study of the small-molecules excreted by the cells under determined conditions [1]. In addition, it can provide useful information on the bacterial uptake or release of nutrients in culture media, which provides useful insights to study bacterial physiology, functional genomics, and strain characterization at molecular level [2,3].

Rhizobia are gram-negative nitrogen-fixing bacteria, belonging to the *Rhizobiaceae* family widespread in soils and employed in agriculture as biofertilizer. Rhizobia form symbiotic associations with leguminous plants promoting their growth through the formation of root nodules, inside which they reduce dinitrogen providing ammonia to their hosts [4]. This interaction is mediated by signaling molecules and metabolic pathways [5], which help in sensing the micro-environmental conditions in the host, and also allow rhizobia to respond to stress conditions and benefit the host by playing a role in immunity [6].

Most rhizobia are endosymbionts of leguminous plants, where they enter into the root and form new organs called nodules in a biochemically orchestrated process. The infection process occurs mainly through two mechanisms: the entrance of rhizobium by a fissure in the root tissue, or mediated by flavonoids and nodulation factors, which is the most common [7]. Once inside the root, the differentiation of the epithelium starts to subsequently form nodules. In addition, a peribacteroid plant-derived membrane is created and rhizobia differentiate into bacteroids, the nitrogen-fixing form of the bacteria. The nitrogen fixation process requires a huge amount of energy obtained from adenosine triphosphate (ATP) molecules, to reduce the nitrogen to ammonia, via the nitrogenase enzyme complex in which several steps of electron transfer occur [8].

These bacteria can survive in soil and rhizosphere [9], and some of them in contaminated soils [10,11]. Thus their interactions are not only restricted to the hosts, but also occur with predators and other rhizobia. This symbiotic association may be host-specific [12]. However, some legumes are nodulated by multiple bacterial strains, thus selection of highly effective ones is important in the development of biofertilizers [13].

Recently, rhizobia have been tested for growth promotion of non-leguminous plants that may be used for feed or biofuels [9]. Rhizobia can establish associations with rice, maize, wheat, and other cereals, sometimes as endophytes, without nodule formation, promoting plant growth [14]. Biofertilizers based on rhizobia are becoming an effective tool for sustainable agriculture of leguminous and non-leguminous plants, by substituting for some agrochemicals. Therefore, the identification of their exo-metabolites is needed because they are crucial in the symbiotic association, communication with other organisms, and as growth substrates in the rhizosphere and soil niche [15,16]. There is also a great lack of knowledge in this area.

Significant advances in nuclear magnetic resonance (NMR) sensitivity by the development of associated tools, such as cryoprobes that increase the sensitivity by around 20-fold, have opened the possibility of identifying and quantifying a broad range of natural compounds [17,18]. It has benefited the emergence of new fields such as microbial metabolomics [19,20], and in particular the study the exometabolomes of bacteria without requiring chemical extractions or derivatizations of the samples [3,21,22,23,24,25]. This non-destructive and highly reproducible technique can detect a wide range of structural diverse compounds at micromolar concentrations [26]. NMR and mass spectrometry (MS) are widely used techniques for metabolomics and exo-metabolomics [27,28,29,30]. However, they both have different analytical strength and weaknesses [27]. We used an 1H-NMR exo-metabolomics approach in this article [19]. This quantitative technique analyzes structurally diverse compounds in a single run at nearly room temperature [20]. In addition, NMR offers enormous benefits in terms of simple sample preparation that is important in microbial metabolomics, as bacterial matrices usually contain compounds that interfere with derivatization [31], sample processing, and analysis [32]. Other benefits of NMR-based exo-metabolomics compared to MS is its nondestructive and nonequilibrium perturbing technique [27], as well as its high reproducibility and quantification power [28].

For many years our focus on rhizobial research has been on the nitrogen-fixing symbionts of *Phaseolus vulgaris* (widely consumed as grains for human nutrition), especially *Rhizobium phaseoli, R. etli, R. tropici* and *R. leucaena*. *Sinorhizobium americanum*, though isolated from acacia plants, is capable of forming nitrogen-fixing nodules in common bean plants. We chose all of them to evaluate if there were convergent excreted molecules due to their sharing a legume host. Exo-metabolite analysis by Nuclear Magnetic Resonance (NMR) is a novel approach in *P. vulgaris* symbiont research.

## 2. Results

### 2.1. Bacterial Growth and Sample Preparation

A growth curve (OD_600_) and colony forming units (CFUs, Figure S1) of the rhizobial strains cultured in liquid minimal medium (MM) at 30 °C and 250 rpm) were used to determine adequate time points for sampling. Based on the data, two time points were selected, one at exponential or late exponential phase (24 h) and the other at stationary phase (50 h). The CFUs of all the strains were above 2 × 10^8^ CFU/mL at both sampling times, except for *R. phaseoli* Ch24-10 at 50 h of growth with 5.0 × 10^7^ CFU/mL. 

### 2.2. Exo-Metabolite Identification by NMR

A total of 37 molecules were detected in the samples by ^1^H NMR, of which nine were from the liquid minimal medium (MM) and 28 corresponded to exo-metabolites solely present in rhizobia extracellular supernatants. The chemical shift of the compounds used for identification purposes are presented in Table S1.

**Figure 1:**
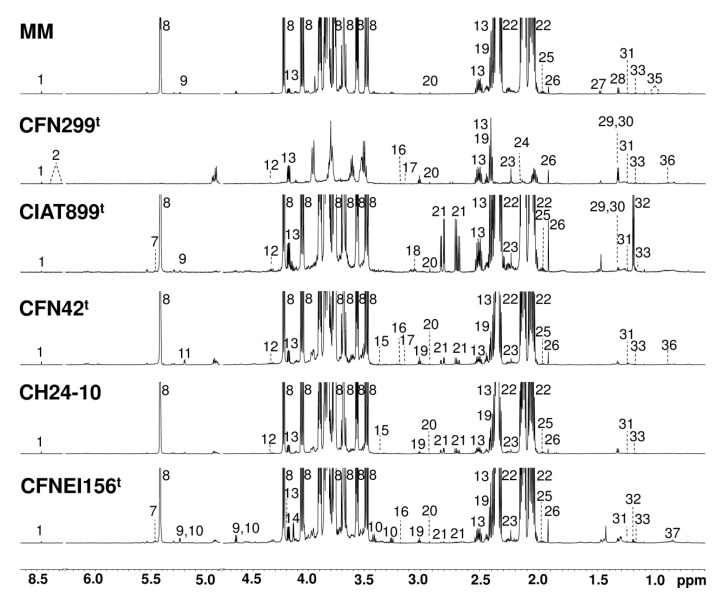
Representative ^1^H nuclear magnetic resonance (NMR) spectra (700 MHz, 298 K, TSP = 1 mM, D_2_O sodium phosphate buffer 0.123 M at pH 7.4) of minimal medium (MM) and rhizobial strain culture supernatants at exponential growth (24 h). Labeled metabolites: 1: Formate, 2: S-Adenosylhomocysteine, 7: Glucose-1-phosphate, 8: Sucrose, 9: Glucose, 10: Maltose, 12: Tartrate, 13: Pyroglutamate, 14: Gluconate, 15: Methanol, 16: Dimethyl sulfone, 17: Malonate, 18: Ornithine, 19: 2-Oxoglutarate, 20: N,N-Dimethylglycine, 21: Aspartate, 22: Glutamate, 23: Acetone, 24: Methionine, 25: Homoserine, 26: Acetate, 27: Alanine, 28: Threonine, 29: Lactate, 30: 3-Hydroxy-3-methylglutarate, 31: 3-Hydroxyisovalerate, 32: 3-Hydroxybutyrate, 33: Ethanol, 35: Valine, 36: 3-Methylglutarate, and 37: Caprylate.

Representative ^1^H NMR spectra for the rhizobial strains and reference culture medium at 24 h of growth are shown in Figure 1.

**Figure 2:**
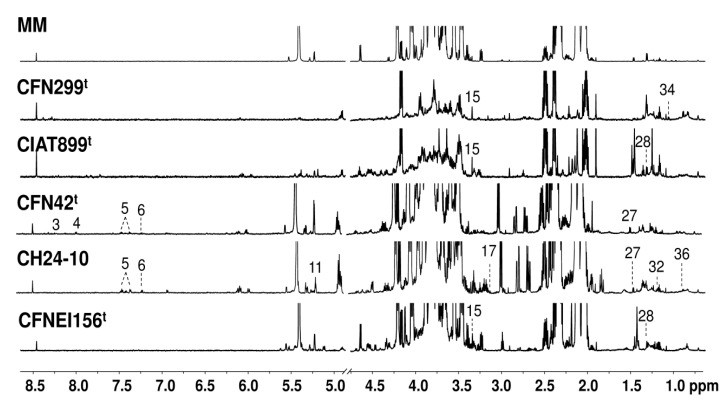
Representative ^1^H NMR spectra (700 MHz, 298K, TSP = 1 mM, D_2_O sodium phosphate buffer 0.123M at pH 7.4) of MM and rhizobial strains culture supernatant at stationary phase (50 h), the compounds that were only detected at this sampling time in comparison to spectra of rhizobial strains at 24 h of growth are shown. Labeled metabolites: 3: Oxypurinol, 4: UDP-glucose, 5: Phenylalanine, 6: Tyrosine, 11: Trehalose, 15: Methanol, 17: Malonate, 27: Alanine, 28: Threonine, 32: 3-Hydroxybutyrate, 34: 3-hydroxyisobutyrate, and 36: 3-Methylglutarate.

The recorded ^1^H NMR spectra of extracellular cultures of the five strains at 50 h exhibited a significant variation of the signals, with a clear decrease of sucrose in some of them. Methanol, and other compounds such as malonate, alanine, and threonine, appear to be present at 50 h but not at 24 h for some strains. Representative spectra of the exo-metabolites and reference MM at 50 h is presented in Figure 2; the numbers represent those molecules that were not present in the spectrum of the same strain at 24 h.

2D-NMR recorded spectra were analyzed with Chenomx (Chenomx Inc., Edmonton, AB, Canada) for accurate assignment and identification of the exo-metabolites. This was exceptionally useful in crowded regions to clarify the multiplicity and number of signals through 2D J-resolved spectra (JRES) spectra, as well as to determine the association between peaks. Lactate, aspartate, sucrose, glutamate, and pyroglutamate were confirmed by the correlation of signals in correlation spectroscopy (COSY) NMR spectra (Figure S2).

### 2.3. Statistical Analysis

**Figure 3:**
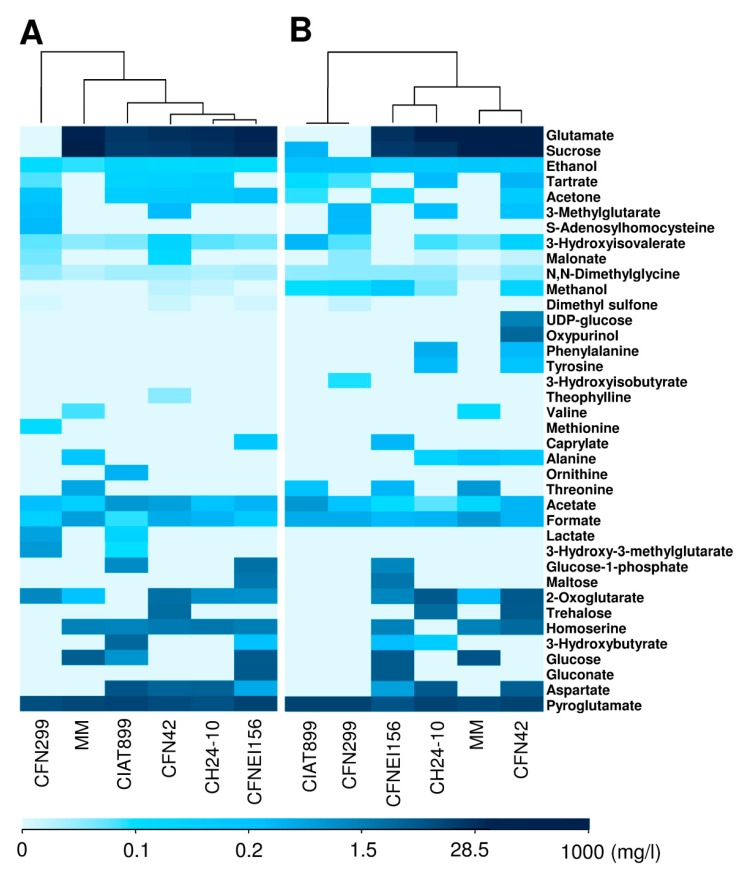
Heatmap and clustering trees based on the concentration profiles of the molecules found in supernatants either produced by rhizobial strains or from the MM (devoid of bacteria) at: (**A**) 24 h and (**B**) 50 h of growth.

Certain molecules appeared and others were not detected or changed their concentration at 50 h in contrast with the NMR data recorded at 24 h of growth. In order to better visualize this, a heatmap with clustering trees on top is presented in Figure 3, which is based on the exo-metabolites concentrations determined by Chenomx (Chenomx Inc.) using ^1^H NMR spectra (Tables S2 and S3).

**Figure 4:**
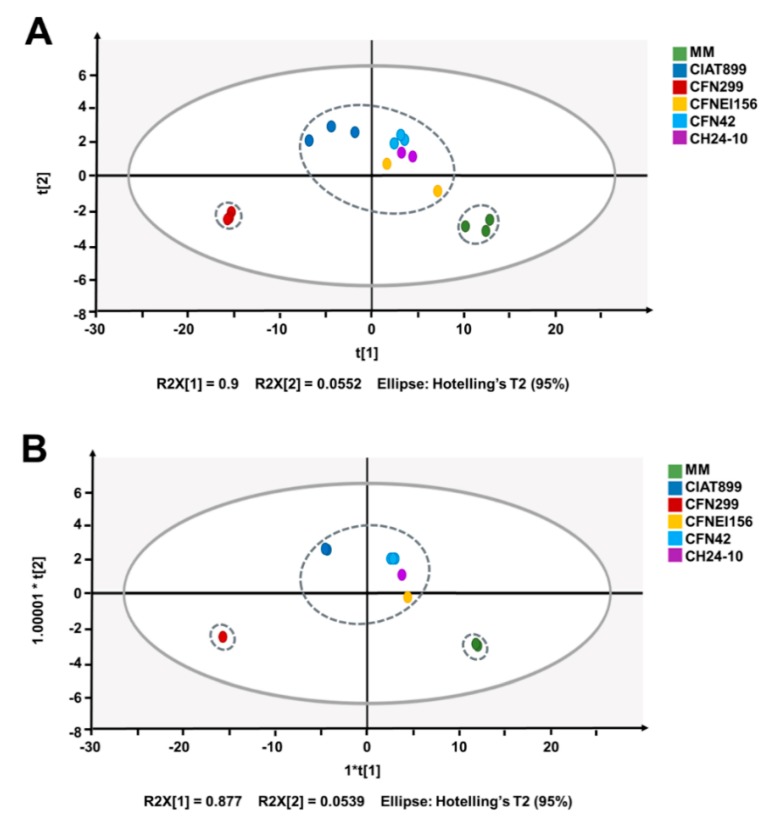
Score plots of (**A**) principal component analysis (PCA) and (**B**) orthogonal partial least squares-discriminant analysis (OPLS-DA) of the ^1^H NMR spectra of the five rhizobial strains and MM.

To evaluate variations between biological replicates and the extracellular medium of the different rhizobial strains according to their NMR spectra, a multivariate analysis was carried out. Principal component analysis (PCA) and orthogonal partial least squares-discriminant analysis (OPLS-DA) of ^1^H NMR spectra showed a clear discrimination between MM (negative-control) and samples (Figure 4B), with a R2X (cum) of 0.981 and 0.999, and Q2 (cum) of 0.957 and 0.841, respectively. The sample corresponding to *R. leucaenae* CFN 299 was different from the others, with the lowest concentration of sucrose and glutamate, which are two of the most concentrated molecules in the rest of the samples (Figure 3).

One-dimensional analysis of variance (ANOVA) showed that significant differences existed in the concentrations of the detected compounds at 24 h, except for ethanol, N,N-dimethylglycine and pyroglutamate, which are suspected to be impurities of the MM. In addition, Tukey’s HSD post-hoc tests with 95% confidence interval were used to evaluate the concentration differences in molecules detected in both MM and bacterial supernatants (Table S4).

## 3. Discussion

The increase in NMR sensitivity allows the study of microbial exo-metabolomes without requirements of extraction or derivatization of the samples. In this research, we identified and quantified the extracellular metabolites of five rhizobial strains by NMR, with the minimum concentration of a detected compound at 0.018 mg/L. Most of the identified compounds were related to fermentative metabolism and stress resistance.

The exo-metabolomic profile of the distinct strains was found to be diverse, but remarkably related strains had a tendency to share common patterns, as observed in *R. phaseoli* and *R. etli* which are closely related species. However this is not observed in *R. tropici* and *R. leucaenae*, which belong to the tropici group, but belong to two different types having significant phenotypic differences [33]. It is notable that *R. leucaenae* exo-metabolites are notably different from the other rhizobial strains, maybe in relation to the optimal growth of the bacteria in the minimal medium that was originally designed to grow the *R. leucaenae* strain CFN299. A further analysis with more strains from each species will help to define if the exo-metabolomic profile is species or strain specific.

The only compound that was excreted by all of the tested rhizobial strains at 24 h was acetone at concentrations ranging from 0.21 to 0.34 mg/L. However, this was not detected or had lower concentrations in most of the strains at 50 h of growth. This product of bacterial fermentation, has also been reported as a microbial carbon source in some gram-positive bacteria [34]. Tartrate, a C4-dicarboxylate, was excreted by all the strains at 24 h except by *Sinorhizobium americanum* CFNEI156. On the other hand, aspartate was released in high amounts (0.74–16.58 mg/L) from all the strains except by *R. leucaenae* CFN299^T^. It has been observed that this amino acid stimulates nitrogen fixation of bacteroids isolated from soybean root nodules [35].

*R. leucaenae* CFN299 was the only strain that totally consumed the glutamate and sucrose provided in the MM at 24 h. Glutamate concentrations in the culture medium after 24 h were 505.73 to 635.38 mg/L, compared to 800.65 mg/L in the MM. In contrast, sucrose concentrations in the culture medium ranged from 275.31 to 626.78 mg/L at 24 h of growth. Besides *R. leucaenae* CFN299, *R. tropici* CIAT 899 was the strain with a low glutamate and sucrose concentration in the final medium.

Ornithine was only detected in the culture supernatant of *R. tropici* CIAT 899^T^ at 24 h but not at 50 h. This finding is interesting as this strain produces ornithine containing membrane lipids, which are involved in symbiotic efficiency and resistance to stress conditions, such as acidity [36,37].

*R. leucaenae* CFN299 was the only strain in which methionine and S-adenosylhomocysteine were detected at 24 h, and the latter was also present at 50 h of growth. In some rhizobial strains, such as *R. etli*, methionine is required for growth and formation of effective nodules [38,39]. This amino acid is also used as a precursor of ethylene in plants, and has numerous benefits in plant growth and development [40]. Furthermore, methionine synthase involved in methionine synthesis [41] is annotated in the genome of *R. leucaenae* (WP_028752452.1). On the other hand, S-adenosylhomocysteine is used in the biosynthesis of the membrane lipid phosphatidylcholine by rhizobia [42].

Trehalose was found in *Bradyrhizobium japonicum* bacteroids [43,44] and in *Phaseolus vulgaris* nodulated plants associated with osmotic stress tolerance [45,46] indicating that trehalose is a product provided to the plant by the bacteria. Trehalose was identified in the culture supernatant of *R. etli* CFN42 at 24 h and the genes encoding enzymes of its biosynthetic pathway have been found in this strain and are likely to be present in most rhizobia [46]. Trehalose was also present in the culture medium of *R. phaseoli* Ch24-10 at 50h. 

3-hydroxybutyrate was present in supernatants of *R. tropici* CIAT899 and *S. americanum* CFNEI156 at 24 h, as well as in *R. phaseoli* Ch24-10 at 50 h of growth. Poly-3-hydroxybutyrate is one of the major carbon storage compounds and affects nitrogen fixation in *Rhizobium etli* [47]. This compound has also been reported to be accumulated in some rhizobia in free-living state [48], and seems to be a potent cryoprotectant metabolite produced by several bacteria [49].

Caprylate, gluconate, and maltose were only detected in the culture supernatant of *S. americanum* CFNEI 156 at 24 h and 50 h of growth. Caprylate is used against envelope viruses [50], its sodium salt is an antifungal agent [51], and currently some production processes of this compound include chain-elongating bacteria [52]. Some bacteria such as *Clostridium kluyveri* can transform acetate and ethanol to short- and medium-chain fatty acids such as butyrate, caproate, and caprylate through chain elongation [53]. This is interesting, as we also found acetate in MM (0.2 mg/L) and in higher concentrations in the culture medium of all strains at 24 h (0.30–1.17 mg/L). Gluconate has been observed to be produced from rhizobium and other bacteria metabolism of glucose by glucose dehydrogenase [54], and is considered responsible for acidifying the rhizosphere [55].

Glucose-1-phosphate was present in CIAT899 and CFNEI156 supernatants at 24 h of growth and only in the latter at 50 h, with a decrease in concentration. Glucose-1-phosphate has been suggested as intermediate of exo-polysaccharide formation in rhizobia [56] and in some maltose-assimilating bacteria [57], which is interesting as maltose production was also found in *S. americanum* CFNEI156.

Malonate was present in *R. leucaenae* CFN299 and *R. etli* CFN42 supernatants at 24 h and at 50 h with a reduction in its concentration, and in *R. phaseoli* Ch24-10 at 50 h of growth. This compound is produced by plants and seems to have an important role in the legume symbiotic-association with rhizobia [58,59], and could be produced by a limited number of bacteria as a result of the degradation of pyrimidines and purines [60].

Dimethyl sulfone was detected in *R. leucaenae* CFN299, *S. americanum* CFNEI156, and *R. etli* CFN42 at 24 h, and only in CFN299 at 50 h. *Sinorhizobium* sp. KT 55 has been reported to use dimethyl sulfone and other compounds as a sole sulfur source, and the bacterium has been proposed as a bioremediator because of its desulfurization activity, and in particular the degradation of benzothiophene, useful in petroleum processing to avoid acid rain [61].

3-methylglutarate was present in *R. leucaenae* CFN299 and *R. etli* CFN42. Besides, 3-hydroxy-3-methylglutarate and lactate were detected in *R. leucaenae* CFN299 and *R. tropici* CIAT 899. Lactate is a byproduct of the carbon metabolism in rhizobia, as well as oxaloacetate, ethanol, malate, succinate, or L-alanine [62]. The gene encoding lactate dehydrogenase, an enzyme involved in producing lactate [63], is present in the genomes of *R. leucaenae* (WP_028754357.1) and *R. tropici* (WP_015341529.1). On the other hand, 3-hydroxy-3-methylglutarate has been proposed as a mediator in the synthesis of polyketides, such as mupirocin in *Pseudomonas*, using acetate as a starting material, with the involvement of other molecules such as S-adenosyl-methionine in the biosynthesis process [64]. This is interesting as it has been hypothesized that polyketides may have a role in the host specify association of rhizobia [65]. Furthermore, we found that the culture medium of *R. leucaenae* CFN299 at 24 h contains acetate, 3-hydroxy-3-methylglutarate, and S-adenosylhomocysteine, which is formed by the demethylation of S-adenosyl methionine. These metabolites could be involved in the formation of polyketides in this strain.

Some compounds were not detected in the culture medium of the strains at 24 h but at 50 h (Figure 2), and vice versa. Among the compounds that were detected at 50 h in contrast with the spectrum of the same strain at 24 h were: oxypurinol, UDP-glucose, phenylalanine, tyrosine, trehalose, methanol, malonate, alanine, threonine, 3-hydroxybutyrate, 3-hydroxyisobutyrate, and 3-methylglutarate. However, further studies are recommended to establish which of these molecules could be released by cellular lysis or through micro-vesicles secreted by rhizobia.

Oxypurinol is produced by CFN42 and maybe a scavenger of the highly reactive hydroxyl radical and a metabolite of allopurinol [66]. These products seem to be released in hypoxic conditions and inhibit the nitrogenase activity in established nodules of cowpea plants, which could be reversed by increasing the oxygen concentration [67]. In *Escherichia coli*, UDP-glucose acts as signaling molecule in the control of the expression of genes related to osmotic regulation and induction of the stationary-phase [68]. Phenylalanine is a precursor of flavonoids biosynthesis in plants [69]. Rhizobia are not tyrosine auxotrophs thus they have all enzymes to produce it. In addition, this amino acid has been described as a precursor in the synthesis of the black pigment melanine in *R. etli* CFN42 [70,71]. In agreement with that, we found this aromatic amino acid in the extracellular medium of CFN42, but also of Ch24-10 at 50 h. In other bacteria, glucose-1-phosphate and UDP-glucose have been reported to be intermediates in the biosynthesis of exopolysaccharides [72]. In addition, UDP-glucose 4-epimerase GalE (WP_012482602.1) and UTP-glucose-1-phosphate uridylyltransferase GalU (WP_041683936.1) have been found in *Rhizobium sp.*, which may be involved in UDP-glucose biosynthesis [73]. Interestingly, all tested rhizobial strains produced methanol at 50 h of growth. This compound has been described as carbon source for the methylotrophic *Methylobacterium* [74].

The compounds consumed, transformed, or degraded by at least one strain at 50 h that were identified in the spectrum of the same strain at 24 h were glutamate, acetone, 3-hydroxyisovalerate, dimethyl sulfone, methionine, ornithine, glucose-1-phosphate, 2-oxoglutarate, 3-hydroxy-3-methylglutarate, lactate, homoserine, 3-hydroxybutyrate, glucose, and aspartate (Figure 3).

According to the heatmap and clustering tree generated in R using the concentrations of identified compounds (Figure 3), as well as the OPLS-DA analysis based on the metabolic footprinting of the strains (Figure 4), rhizobial strains exhibit commonalities and differences between them, not only in terms of the concentration ranges but in the compounds consumed and generated. Such differences between strains may be conferring them distinct phenotypical features. Some of the identified compounds are related to carbon metabolism, stress resistance, and symbiotic efficiency, among others. Further studies on the role of the released compounds of rhizobia in symbiosis, plant growth promotion, or bioremediation are needed. This first study of the exo-metabolites of rhizobia by 1H NMR opens the possibility to use similar approaches to identify exo-metabolites from rhizobia using different culture media composition or oxygen levels. A joint transcriptomic analysis would allow us to correlate bacterial gene expression with metabolite production.

## 4. Materials and Methods

### 4.1. Bacterial Growth and Sample Preparation

The nitrogen-fixing rhizobial strains used in this study were *Rhizobium etli* CFN 42^T^, *Rhizobium leucaenae* CFN 299^T^, *Rhizobium tropici* CIAT 899^T^, *Rhizobium phaseoli* Ch24-10, and *Sinorhizobium americanum* CFNEI 156^T^.

Each rhizobial strain was grown separately on peptone yeast (PY) agar (peptone, 5 g; yeast extract, 3 g; CaCl_2_, 0.6 g; agar, 18 g per L) for 2 days. They were precultured by triplicate in 30 mL of MM: K_2_HPO_4_ 3.8 g/L, KH_2_PO_4_ 3 g/L, sucrose 1 g/L, glutamate 1 g/L, MgSO_4_·7H_2_O 0.1 g/L, CaCl_2_ 0.1 g/L, H_3_BO_3_ 2.86 mg/L, ferric citrate 5 mg/L, MnSO_4_·4H_2_O 2.03 mg/L, ZnSO_4_·7H_2_O 0.22 mg/L, CuSO_4_·5H_2_O 0.08 mg/L and Na_2_MoO_4_·H2O 0.08 mg/L for 48 h at 30 °C with continuous shaking. Three biological replicates of final bacterial cultures were made by diluting the precultures 1:20 with fresh MM (OD_600_ ~ 0.1) to a final volume of 30 mL, and they were kept at 30 °C with continuous shaking. In order to select the sampling times, growth curves based on the optical density OD_600_ and colony forming units (CFU) were determined at 8 h, 24 h, and 50 h.

The rhizobial strains were precultured and cultured by triplicates following the previous protocol, and samples were taken at 24 h (exponential phase) and 50 h (stationary phase). A volume of 1.5 mL of the liquid bacterial cultures and MM incubated under the same conditions (control) were transferred to eppendorf tubes and centrifuged during 10 min at 4000 g, 1 mL of the supernatant was taken and lyophilized to dryness.

### 4.2. Exo-Metabolite Identification by NMR

The equipment we used was a 700 MHz NMR spectrometer equipment CryoProbe (Bruker, Fällanden, Switzerland) which improves the sensitivity and provides highly reproducible data, useful for multivariate statistical methods [31].

A total of 24 samples were processed: three biological replicates of the culture supernatants of five rhizobial strains and MM at 24 h, and one replicate of each at 50 h of incubation. The freeze-dried extracellular cultures and MM were separately dissolved in 600 µL of D_2_O sodium phosphate buffer 0.123M at pH 7.4 with 1 mM of sodium salt of trimethylsilylpropionic acid (TSP) as internal standard. NMR analysis was carried out on an Avance III HD 700 spectrometer at 298 K with a ^1^H frequency of 699.95 MHz (Bruker, Billerica, MA, USA) equipped with a 5-mm z-axis gradient TCI cryogenic probe.

1D ^1^H NMR spectra were acquired by using the standard NOESY-1D pulse sequence (Bruker program *noesypr1d*) that allows water suppression maintaining the intensity of most of the remaining signals [75]. Water resonance was irradiated during relaxation delay (RD) of 4.0 s and mixing time of 10 ms. Each spectrum consisted of 256 scans with 14 kHz spectral width, 64 k data points and an acquisition time of 2.3 s. An exponential line-broadening factor of 0.3 Hz was applied to the free induction decays (FID) before Fourier transformation.

Additionally, 2D-NMR experiments were carried out on representative samples to confirm chemical shift assignments, including JRES, COSY, total correlation spectroscopy (TOCSY), heteronuclear multiple bond correlation (HMBC), heteronuclear single quantum coherence spectroscopy (HSQC), and diffusion ordered NMR spectroscopy (DOSY).

NMR data were recorded using Topspin v 3.5.6 and processed using MestReNova v. 12.0 (MestreLab Research SL., Santiago de Compostela, Spain). Phase and baseline were corrected manually, and TSP chemical shift referenced to 0.000 ppm.

^1^H NMR raw data were used as input to Chenomx NMR Suite v. 8.31 (Chenomx Inc., Edmonton, AB, Canada) for identification and quantification of the extracellular metabolites in the rhizobial strains and the molecules present in the MM in each of the biological replicates at 24 h and 50 h of growth. Chenomx Processor was used to manually adjust the phase and baseline of each spectrum, with the following parameters, TSP concentration: 1 mM, pH: 7.4 ± 0.50 and line broadening: 0.30 Hz. Water regions were removed and the chemical shape indicator (CSI) adjusted to the TSP 1 mM peak. Once prepared, these spectra were charged to Chenomx Profiler for the identification of small-molecules based on the location of their resonances on the 700-MHz 1H-NMR spectra. This software is linked to a database containing the NMR spectral signatures of more than 250 compounds.

### 4.3. Statistical Analysis

Based on the quantification of the compounds in the extracellular culture medium by ^1^H NMR and Chenomx software v. 8.31 (Chenomx Inc., Edmonton, AB, Canada), heatmaps for exo-metabolites at 24 h and 50 h were built using the “heatmap.2” function of the gplots 3.0.1 library [76] of R version 3.5.1. [77]. Due to the broad dispersion of the data, the color breaks were set up according to quantiles, in which dark blue represent those molecules with the highest concentration. Dendrograms were added on top of the heatmaps for comparison purposes between strains.

Spectra of rhizobium strains and their replicates at 24 h were reduced to ASCII format. These were previously processed by MestreNova software v. 12.0 (MestreLab Research SL., Santiago de Compostela, Spain) through phase and baseline correction, normalization at TSP (0.000 ppm), water region suppression (4.730–4880 ppm) and superposition of the spectra. In the region from −0.5 to 10.0 ppm, 1H-NMR spectra were split in regions of width: 0.06 ppm, giving a total of 258 spectral segments per spectrum (bins) by using Chenomx software v. 8.31 (Chenomx Inc., Edmonton, AB, Canada). These data were used for multivariate analysis (MVDA) with SIMCA 14.1.0.2047 software (MKS Umetrics, Malmö, Sweden).

To identify differences in the exo-metabolomics profiles of the five rhizobial strains, principal component analysis (PCA), orthogonal partial least squares-discriminant analysis (OPLS-DA), and score plots of the binned spectral data were generated in SIMCA 14.1.0.2047 software (MKS Umetrics, Sweden) by pareto scaling. Data were organized in six classes—five strains and the control—and replicates were considered as observations of the same group. In addition, ANOVA and Tukey’s HSD post-hoc tests were performed in R version 3.5.1. [77] to evaluate if significant differences exist between the concentrations of the molecules detected in MM and bacterial supernatants. Outliers were removed.

## Supplementary Materials

The following are available online at https://www.mdpi.com/2218-1989/9/6/105/s1, Figure S1: (a) Growth curve based on optical density (OD_600_) at logarithmic scale and (b) colony formation units; Figure S2: Representative ^1^H 2-D COSY NMR spectra of the (a) MM, and the culture medium of the strains (b) CFN299 and (c) ch24-10; Table S1: Chemical shifts of the compounds identified by ^1^H NMR (700 MHz, 298K, TSP = 1 mM, D_2_O sodium phosphate buffer 0.123M at pH 7.4). TSP was used as internal standard and referenced to chemical shift 0.000 ppm; Table S2: Concentrations of the detected molecules by ^1^H NMR (700 MHz, 298K, TSP = 1 mM, D_2_O sodium phosphate buffer 0.123M at pH 7.4) in the MM and bacterial culture supernatants at 24 h of incubation; Table S3: Concentrations of the detected molecules by ^1^H NMR (700 MHz, 298K, TSP = 1 mM, D_2_O sodium phosphate buffer 0.123M at pH 7.4) in the MM and bacterial culture supernatants at 50 h of incubation. Table S4. Results of one-way ANOVA (p-values) and Tukey´s HSD post hoc test with 95% confidence interval (p-values adjusted) calculated in R.

## References

[1] Mapelli V., Olsson L., Nielsen J. (2008). Metabolic footprinting in microbiology: Methods and applications in functional genomics and biotechnology. Trends Biotechnol..

[2] Behrends V., Williams H.D., Bundy J.G., Filloux A., Ramos J.-L. (2014). Metabolic footprinting: Extracellular metabolomic analysis. Pseudomonas Methods and Protocols.

[3] Palama T.L., Canard I., Rautureau G.J., Mirande C., Chatellier S., Elena-Herrmann B. (2016). Identification of bacterial species by untargeted NMR spectroscopy of the exo-metabolome. Analyst.

[4] Guinel F.C. (2015). Ethylene, a hormone at the center-stage of nodulation. Front. Plant Sci..

[5] Peix A., Velazquez E., Silva L.R., Mateos P.F. (2010). Key Molecules Involved in Beneficial Infection Process in Rhizobia-Legume Symbiosis.

[6] Gibson K.E., Kobayashi H., Walker G.C. (2008). Molecular determinants of a symbiotic chronic infection. Annual Review of Genetics.

[7] Poole P., Ramachandran V., Terpolilli J. (2018). Rhizobia: From saprophytes to endosymbionts. Nat. Rev. Microbiol..

[8] Mitsch M.J., diCenzo G.C., Cowie A., Finan T.M. (2018). Succinate transport is not essential for symbiotic nitrogen fixation by *Sinorhizobium meliloti* or *Rhizobium leguminosarum*. Appl. Environ. Microbiol..

[9] Janczarek M. (2011). Environmental signals and regulatory pathways that influence exopolysaccharide production in rhizobia. Int. J. Mol. Sci..

[10] Arora N.K., Khare E., Singh S., Maheshwari D.K. (2010). Effect of Al and heavy metals on enzymes of nitrogen metabolism of fast and slow growing rhizobia under explanta conditions. World J. Microbiol. Biotechnol..

[11] Keum Y.S., Seo J.S., Li Q.X., Kim J.H. (2008). Comparative metabolomic analysis of *Sinorhizobium* sp. C4 during the degradation of phenanthrene. Appl. Microbiol. Biotechnol..

[12] Janczarek M., Rachwal K., Marzec A., Grzadziel J., Palusinska-Szysz M. (2015). Signal molecules and cell-surface components involved in early stages of the legume-rhizobium interactions. Appl. Soil Ecol..

[13] Marczak M., Mazur A., Koper P., Zebracki K., Skorupska A. (2017). Synthesis of rhizobial exopolysaccharides and their importance for symbiosis with legume plants. Genes.

[14] Mia M.B., Shamsuddin Z. (2010). Rhizobium as a crop enhancer and biofertilizer for increased cereal production. Afr. J. Biotechnol..

[15] Gemperline E., Jayaraman D., Maeda J., Ane J.M., Li L.J. (2015). Multifaceted investigation of metabolites during nitrogen fixation in medicago via high resolution MALDI-MS Imaging and ESI-MS. J. Am. Soc. Mass Spectrom..

[16] Jacoby R.P., Martyn A., Kopriva S. (2018). Exometabolomic profiling of bacterial strains as cultivated using arabidopsis root extract as the sole carbon source. Mol. Plant Microbe Interact..

[17] Dear G.J., Roberts A.D., Beaumont C., North S.E. (2008). Evaluation of preparative high performance liquid chromatography and cryoprobe-nuclear magnetic resonance spectroscopy for the early quantitative estimation of drug metabolites in human plasma. J. Chromatogr. B.

[18] Molinski T.F. (2009). Nanomole-scale natural products discovery. Curr. Opin. Drug Discov. Dev..

[19] Tang J. (2011). Microbial metabolomics. Curr. Genom..

[20] Mashego M.R., Rumbold K., De Mey M., Vandamme E., Soetaert W., Heijnen J.J. (2007). Microbial metabolomics: Past, present and future methodologies. Biotechnol. Lett..

[21] Puntus I., Sakharovsky V., Filonov A., Boronin A. (2005). Surface activity and metabolism of hydrocarbon-degrading microorganisms growing on hexadecane and naphthalene. Process Biochem..

[22] Lee J.-E., Hwang G.-S., Lee C.-H., Hong Y.-S. (2009). Metabolomics reveals alterations in both primary and secondary metabolites by wine bacteria. J. Agric. Food Chem..

[23] Murovec B., Makuc D., Repinc S.K., Prevoršek Z., Zavec D., Šket R., Pečnik K., Plavec J., Stres B. (2018). ^1^H NMR metabolomics of microbial metabolites in the four MW agricultural biogas plant reactors: A case study of inhibition mirroring the acute rumen acidosis symptoms. J. Environ. Manag..

[24] Hoerr V., Duggan G.E., Zbytnuik L., Poon K.K., Große C., Neugebauer U., Methling K., Löffler B., Vogel H.J. (2016). Characterization and prediction of the mechanism of action of antibiotics through NMR metabolomics. BMC Microbiol..

[25] Chen T., Sheng J., Fu Y., Li M., Wang J., Jia A.-Q. (2017). ^1^H NMR-based global metabolic studies of pseudomonas aeruginosa upon exposure of the quorum sensing inhibitor resveratrol. J. Proteome Res..

[26] Griffith C.M., Morgan M.A., Dinges M.M., Mathon C., Larive C.K. (2018). Metabolic profiling of chloroacetanilide herbicides in earthworm coelomic fluid using 1H NMR and GC-MS. J. Proteome Res..

[27] Beckonert O., Keun H.C., Ebbels T.M., Bundy J., Holmes E., Lindon J.C., Nicholson J.K. (2007). Metabolic profiling, metabolomic and metabonomic procedures for NMR spectroscopy of urine, plasma, serum and tissue extracts. Nat. Protoc..

[28] Markley J.L., Brüschweiler R., Edison A.S., Eghbalnia H.R., Powers R., Raftery D., Wishart D.S. (2017). The future of NMR-based metabolomics. Curr. Opin. Biotechnol..

[29] Baharum S.N., Azizan K.A. (2018). Metabolomics in systems biology. Omics Applications for Systems Biology.

[30] Wishart D.S. (2008). Quantitative metabolomics using NMR. TrAC Trends Anal. Chem..

[31] Pan Z., Raftery D. (2007). Comparing and combining NMR spectroscopy and mass spectrometry in metabolomics. Anal. Bioanal. Chem..

[32] Koek M.M., Muilwijk B., van der Werf M.J., Hankemeier T. (2006). Microbial metabolomics with gas chromatography/mass spectrometry. Anal. Chem..

[33] Ramírez-Puebla S.T., Hernández M.A.R., Ruiz G.G., Ormeño-Orrillo E., Martinez-Romero J.C., Servín-Garcidueñas L.E., Núñez-de la Mora A., Amescua-Villela G., Negrete-Yankelevich S., Martínez-Romero E. (2018). Nodule bacteria from the cultured legume *Phaseolus dumosus* (belonging to the *Phaseolus vulgaris* cross-inoculation group) with common tropici phenotypic characteristics and symbiovar but distinctive phylogenomic position and chromid. Syst. Appl. Microbiol..

[34] Taylor D.G., Trudgill P.W., Cripps R.E., Harris P.R. (1980). The microbial metabolism of acetone. Microbiology.

[35] Kouchi H., Fukai K., Kihara A. (1991). Metabolism of glutamate and aspartate in bacteroids isolated from soybean root nodules. Microbiology.

[36] Rojas-Jiménez K., Sohlenkamp C., Geiger O., Martínez-Romero E., Werner D., Vinuesa P. (2005). A ClC chloride channel homolog and ornithine-containing membrane lipids of *Rhizobium tropici* CIAT899 are involved in symbiotic efficiency and acid tolerance. Mol. Plant Microbe Interact..

[37] Vences-Guzmán M.Á., Guan Z., Ormeño-Orrillo E., González-Silva N., López-Lara I.M., Martínez-Romero E., Geiger O., Sohlenkamp C. (2011). Hydroxylated ornithine lipids increase stress tolerance in *Rhizobium tropici* CIAT899. Mol. Microbiol..

[38] Taté R., Riccio A., Caputo E., laccarino M., Patriarca E.J. (1999). The *Rhizobium etli* metZ gene is essential for methionine biosynthesis and nodulation of *Phaseolus vulgaris*. Mol. Plant Microbe Interact..

[39] Watson R.J., Heys R., Martin T., Savard M. (2001). *Sinorhizobium meliloti* Cells Require Biotin and either Cobalt or Methionine for Growth. Appl. Environ. Microb..

[40] Szilagyi-Zecchin V.J., Mógor Á.F., Figueiredo G.G.O., Singh D.P., Singh H.B., Prabha R. (2016). Strategies for characterization of agriculturally important bacteria. Microbial Inoculants in Sustainable Agricultural Productivity: Vol. 1: Research Perspectives.

[41] Barra L., Fontenelle C., Ermel G., Trautwetter A., Walker G.C., Blanco C. (2006). Interrelations between glycine betaine catabolism and methionine biosynthesis in *Sinorhizobium meliloti* strain 102F34. J. Bacteriol..

[42] de Rudder K.E., Sohlenkamp C., Geiger O. (1999). Plant-exuded choline is used for rhizobial membrane lipid biosynthesis by phosphatidylcholine synthase. J. Biol. Chem..

[43] Vauclare P., Bligny R., Gout E., Widmer F. (2013). An overview of the metabolic differences between Bradyrhizobium japonicum 110 bacteria and differentiated bacteroids from soybean (Glycine max) root nodules: An in vitro 13C-and 31P-nuclear magnetic resonance spectroscopy study. FEMS Microbiol. Lett..

[44] Streeter J.G. (1985). Accumulation of alpha, alpha-trehalose by Rhizobium bacteria and bacteroids. J. Bacteriol..

[45] Farías-Rodríguez R., Mellor R.B., Arias C., Peña-Cabriales J.J. (1998). The accumulation of trehalose in nodules of several cultivars of common bean (Phaseolus vulgaris) and its correlation with resistance to drought stress. Physiol. Plantarum.

[46] McIntyre H.J., Davies H., Hore T.A., Miller S.H., Dufour J.-P., Ronson C.W. (2007). Trehalose biosynthesis in Rhizobium leguminosarum bv. trifolii and its role in desiccation tolerance. Appl. Environ. Microbiol..

[47] Wang C.X., Saldanha M., Sheng X.Y., Shelswell K.J., Walsh K.T., Sobral B.W.S., Charles T.C. (2007). Roles of poly-3-hydroxybutyrate (PHB) and glycogen in symbiosis of *Sinorhizobium meliloti* with *Medicago* sp.. Microbiology.

[48] Kim S.A., Copeland L. (1997). Acetyl coenzyme A acetyltransferase of *Rhizobium* sp. (Cicer) strain CC 1192. Appl. Environ. Microb..

[49] Obruca S., Sedlacek P., Krzyzanek V., Mravec F., Hrubanova K., Samek O., Kucera D., Benesova P., Marova I. (2016). Accumulation of poly (3-hydroxybutyrate) helps bacterial cells to survive freezing. PLoS ONE.

[50] Korneyeva M., Hotta J., Lebing W., Rosenthal R., Franks L., Petteway S. (2002). Enveloped virus inactivation by caprylate: A robust alternative to solvent-detergent treatment in plasma derived intermediates. Biologicals.

[51] Alsaeed T.I. (2015). Antibacterial eFFICacy and Discoloration Effect of a Novel Intracanal Antibiotic Dressing. Ph.D. Thesis.

[52] Angenent L.T., Kucek L. (2017). Methods of and Systems for Producing Caprylic Acid And/Or Caprylate. US Patents.

[53] Reddy M.V., Mohan S.V., Chang Y.-C. (2018). Medium-Chain Fatty Acids (MCFA) Production Through Anaerobic Fermentation Using *Clostridium kluyveri*: Effect of Ethanol and Acetate. Appl. Biochem. Biotech..

[54] Van Schie B., De Mooy O., Linton J., Van Dijken J., Kuenen J. (1987). PQQ-dependent production of gluconic acid by Acinetobacter, Agrobacterium and Rhizobium species. Microbiology.

[55] Giles C.D., Hsu P.-C., Richardson A.E., Hurst M.R., Hill J.E. (2015). The role of gluconate production by *Pseudomonas spp.* in the mineralization and bioavailability of calcium–phytate to Nicotiana tabacum. Can. J. Microbiol..

[56] Skorupska A., Janczarek M., Marczak M., Mazur A., Król J. (2006). Rhizobial exopolysaccharides: Genetic control and symbiotic functions. Microb. Cell Fact..

[57] Sjöberg A., Hahn-Hägerdal B. (1989). β-Glucose-1-phosphate, a possible mediator for polysaccharide formation in maltose-assimilating *Lactococcus lactis*. Appl. Environ. Microb..

[58] Kim Y.-S. (2002). Malonate metabolism: Biochemistry, molecular biology, physiology, and industrial application. BMB Rep..

[59] An J.H., Lee H.Y., Ko K.N., Kim E.-S., Kim Y.S. (2002). Symbiotic effects of *deltamatB Rhizobium leguminosarum* bv. *trifolii* mutant on clovers. Mol. Cells.

[60] Vogels G.v.d., Van der Drift C. (1976). Degradation of purines and pyrimidines by microorganisms. Bacteriol. Rev..

[61] Tanaka Y., Onaka T., Matsui T., Maruhashi K., Kurane R. (2001). Desulfurization of benzothiophene by the gram-negative bacterium, *Sinorhizobium* sp. KT55. Curr. Microbiol..

[62] Stowers M.D. (1985). Carbon metabolism in Rhizobium species. Ann. Rev. Microbiol..

[63] Trinchant J.C., Rigaud J. (1974). Lactate dehydrogenase from *Rhizobium.* purification and role in indole metabolism. Physiol. Plantarum.

[64] El-Sayed A.K., Hothersall J., Cooper S.M., Stephens E., Simpson T.J., Thomas C.M. (2003). Characterization of the mupirocin biosynthesis gene cluster from *Pseudomonas fluorescens* NCIMB 10586. Chem. Biol..

[65] Hopwood D.A., Sherman D.H. (1990). Molecular genetics of polyketides and its comparison to fatty acid biosynthesis. Annu. Rev. Genet..

[66] Moorhouse P.C., Grootveld M., Halliwell B., Quinlan J.G., Gutteridge J.M. (1987). Allopurinol and oxypurinol are hydroxyl radical scavengers. FEBS Lett..

[67] Atkins C.A., Sanford P.J., Storer P.J., Pate J.S. (1988). Inhibition of nodule functioning in cowpea by a xanthine oxidoreductase inhibitor, allopurinol. Plant Physiol..

[68] Böhringer J., Fischer D., Mosler G., Hengge-Aronis R. (1995). UDP-glucose is a potential intracellular signal molecule in the control of expression of sigma S and sigma S-dependent genes in *Escherichia coli*. J. Bacteriol..

[69] Recourt K., van Tunen A.J., Mur L.A., van Brussel A.A., Lugtenberg B.J., Kijne J.W. (1992). Activation of flavonoid biosynthesis in roots of *Vicia sativa* subsp. *nigra* plants by inoculation with Rhizobium leguminosarum biovar viciae. Plant Mol. Biol..

[70] Santos C.N.S., Stephanopoulos G. (2008). Melanin-based high-throughput screen for L-tyrosine production in *Escherichia coli*. Appl. Environ. Microb..

[71] Cubo M.T., Buendia-Claveria A.M., Beringer J.E., Ruiz-Sainz J.E. (1988). Melanin production by Rhizobium strains. Appl. Environ. Microb..

[72] Boels I.C., Ramos A., Kleerebezem M., de Vos W.M. (2001). Functional analysis of the *Lactococcus lactis galU* and *galE* genes and their impact on sugar nucleotide and exopolysaccharide biosynthesis. Appl. Environ. Microb..

[73] Audy J., Labrie S., Roy D., LaPointe G. (2010). Sugar source modulates exopolysaccharide biosynthesis in *Bifidobacterium longum* subsp. *longum* CRC 002. Microbiology.

[74] Bourque D., Pomerleau Y., Groleau D. (1995). High-cell-density production of poly-β-hydroxybutyrate (PHB) from methanol by *Methylobacterium extorquens*: Production of high-molecular-mass PHB. Appl. Microbiol. Biotechnol..

[75] Le Guennec A., Tayyari F., Edison A.S. (2017). Alternatives to nuclear overhauser enhancement spectroscopy presat and carr–purcell–meiboom–gill presat for NMR-based metabolomics. Anal. Chem..

[76] Warnes G., Bolker B., Bonebakker L., Gentleman R., Huber W., Liaw A. (2016). Gplots: Various R Programming Tools for Plotting Data. https://rdrr.io/cran/gplots/.

[77] Team R.C. R: A Language and Environment for Statistical Computing. https://www.gbif.org/tool/81287/r-a-language-and-environment-for-statistical-computing.

