# Supplementary material for: Exo-Metabolites of Phaseolus vulgaris-Nodulating Rhizobial Strains
Source: Metabolites. 2019 May 30;9(6):105. doi: 10.3390/metabo9060105 (PMC6630823; doi:10.3390/metabo9060105)
Supplement: Supplementary file 1 [file metabolites-09-00105-s001.zip › TableS1.pdf]

Table S1. Chemical shifts of the compounds identified by ^{1}H NMR (700 MHz, 298K, TSP=1mM, D_2O sodium phosphate buffer 0.123M at pH 7.4). TSP was used as internal standard and referenced to chemical shift 0.000 ppm.

#	Molecule	^{1}H NMR Chemical shifts(ppm)
1	Formate	8.46 (s).
2	S-Adenosylhomocysteine	8.38 (s), 8.28 (s).
3	Oxypurinol	8.21 (s).
4	UDP-glucose	7.96 (d, *J* = 8.4 Hz).
5	Phenylalanine	7.43 (t, J=7.2 Hz), 7.34 (d, J=7.6 Hz).
6	Tyrosine	7.21-7.19 (m), 6.93-6.89 (m).
7	Glucose-1-phosphate	5.46 (dd, *J* = 7.4, 3.5 Hz).
8	Sucrose	5.42 (d, *J* = 3.9 Hz), 4.22 (d, *J* = 8.8 Hz), 4.06 (t, *J* = 8.6 Hz), 3.92 – 3.83 (m), 3.69 (d, *J* = 1.9 Hz), 3.57 (dd, *J* = 10.0, 3.9 Hz), 3.48 (t, *J* = 9.5 Hz, 0H)
9	Glucose	5.24 (d, *J* = 3.7 Hz), 4.65 (d, *J* = 8.0 Hz).
10	Maltose	5.24 (d, *J* = 3.7 Hz), 4.65 (d, *J* = 8.0 Hz), 3.31 – 3.28 (m).
11	Trehalose	5.20 (d, *J* = 3.7 Hz).
12	Tartrate	4.35 (s).
13	Pyroglutamate	4.18 (dd, *J* = 9.0, 5.8 Hz), 2.51 (dtd, *J* = 12.9, 9.1, 6.8 Hz).
14	Gluconate	4.14 (d, *J* = 3.7 Hz).
15	Methanol	3.36 (s).
16	Dimethyl sulfone	3.16 (s).
17	Malonate	3.14 (s).
18	Ornithine	3.07-3.04 (m).
19	2-Oxoglutarate	3.01 (t, *J* = 6.9 Hz).
20	N,N-Dimethylglycine	2.92 (s).
21	Aspartate	2.82 (dd, *J* = 17.5, 3.8 Hz), 2.69 (dd, *J* = 17.5, 8.7 Hz).
22	Glutamate	2.35 (m), 2.14 (dddd, *J* = 15.2, 8.4, 7.2, 4.8 Hz), 2.07(m).
23	Acetone	2.24 (s).
24	Methionine	2.16 (s).
25	Homoserine	1.97 (dq, *J* = 14.7, 7.6 Hz).
26	Acetate	1.92 (s).
27	Alanine	1.49 (d, *J* = 7.3 Hz).
28	Threonine	1.33 (d, *J* = 6.9 Hz).
29	Lactate	1.33 (d, *J*= 6.9 Hz).
30	3-Hydroxy-3-methylglutarate	1.33 (s).
31	3-Hydroxyisovalerate	1.25 (s).
32	3-Hydroxybutyrate	1.21 (d, *J* = 6.6 Hz).
33	Ethanol	1.19 (t, *J* = 7.1 Hz).
34	3-hydroxyisobutyrate	1.07 (d, *J* = 7.1 Hz).
35	Valine	1.05 (d, *J* = 7.1 Hz), 1.00 (d, *J* = 7.0 Hz).
36	3-Methylglutarate	0.91 (d, *J* = 6.6 Hz).
37	Caprylate	0.87 (t, *J* = 7.0 Hz).
