# Supplementary material for: Exo-Metabolites of Phaseolus vulgaris-Nodulating Rhizobial Strains
Source: Metabolites. 2019 May 30;9(6):105. doi: 10.3390/metabo9060105 (PMC6630823; doi:10.3390/metabo9060105)
Supplement: Supplementary file 1 [file metabolites-09-00105-s001.zip › TableS4.pdf]

Table S4. Results of one-way ANOVA (p-values) and Tukey´s HSD post hoc test with 95% confidence interval (p-values adjusted) calculated in R.(a) compounds solely present in MM, (b) chemicals found in MM and bacterial supernatants with no statistically significant difference in their concentrations, and (c) exo-metabolites not detected in MM.

Molecules	p-values	p-values adjusted				
		MM/CFN299	MM/CIAT899	MM/CFNEI156	MM/CFN42	MM/CH24-10
Glutamate	5.48×10^{-10}	2.80×10^{-10}	4.60×10^{-06}	1.78×10^{-03}	4.26×10^{-04}	1.07×10^{-03}
Sucrose	2.04×10^{-07}	9.51×10^{-08}	1.88×10^{-06}	1.62×10^{-03}	3.82×10^{-05}	4.13×10^{-04}
3-Hydroxyisovalerate	1.77×10^{-03}	7.88×10^{-01}	9.99×10^{-01}	9.20×10^{-01}	1.58×10^{-03}	7.90×10^{-01}
2-Oxoglutarate	4.20×10^{-07}	1.45×10^{-03}	7.14×10^{-01}	1.64×10^{-02}	7.00×10^{-07}	1.54×10^{-02}
Homoserine	9.78×10^{-10}	9.15×10^{-09}	6.45×10^{-01}	2.81×10^{-01}	1.37×10^{-02}	3.83×10^{-04}
Glucose	1.79×10^{-09}	3.04×10^{-08}	1.75×10^{-07}	3.61×10^{-01}	3.04×10^{-08}	9.30×10^{-08}
Formate	8.47×10^{-04}	2.99×10^{-03}	1.30×10^{-03}	1.06×10^{-02}	5.49×10^{-01}	2.29×10^{-01}
Acetate	8.46×10^{-04}	8.49×10^{-01}	8.73×10^{-04}	1.97×10^{-01}	2.23×10^{-02}	9.89×10^{-01}
Threonine(a)	2.37×10^{-11}	-	-	-	-	-
Alanine(a)	5.87×10^{-13}	-	-	-	-	-
Valine(a)	3.77×10^{-10}	-	-	-	-	-
Ethanol(b)	1.58×10^{-01}	-	-	-	-	-
N,N-Dimethylglycine(b)	7.38×10^{-02}	-	-	-	-	-
Pyroglutamate(b)	1.44×10^{-01}	-	-	-	-	-
Trehalose(c)	3.90×10^{-23}	-	-	-	-	-

3-Hydroxy-3-methylglutarate[c]	1.16×10^{-20}	-	-	-	-	-
Maltose[c]	1.87×10^{-14}	-	-	-	-	-
Malonate[c]	5.41×10^{-12}	-	-	-	-	-
Glucose-1-phosphate[c]	3.57×10^{-11}	-	-	-	-	-
Aspartate[c]	4.69×10^{-11}	-	-	-	-	-
3-Methylglutarate[c]	4.03×10^{-08}	-	-	-	-	-
Tartrate[c]	3.06×10^{-07}	-	-	-	-	-
Gluconate[c]	5.50×10^{-07}	-	-	-	-	-
S-Adenosylhomocysteine[c]	9.50×10^{-07}	-	-	-	-	-
Methanol[c]	2.60×10^{-06}	-	-	-	-	-
3-Hydroxybutyrate[c]	4.38×10^{-06}	-	-	-	-	-
Ornithine[c]	8.97×10^{-06}	-	-	-	-	-
Methionine[c]	1.78×10^{-05}	-	-	-	-	-
Dimethyl sulfone[c]	1.97×10^{-05}	-	-	-	-	-
Caprylate[c]	2.39×10^{-05}	-	-	-	-	-
Acetone[c]	1.47×10^{-03}	-	-	-	-	-
Lactate[c]	2.13×10^{-02}	-	-	-	-	-
