# Supplementary figures and images for: Exo-Metabolites of Phaseolus vulgaris-Nodulating Rhizobial Strains
Source: Metabolites. 2019 May 30;9(6):105. doi: 10.3390/metabo9060105 (PMC6630823; doi:10.3390/metabo9060105)

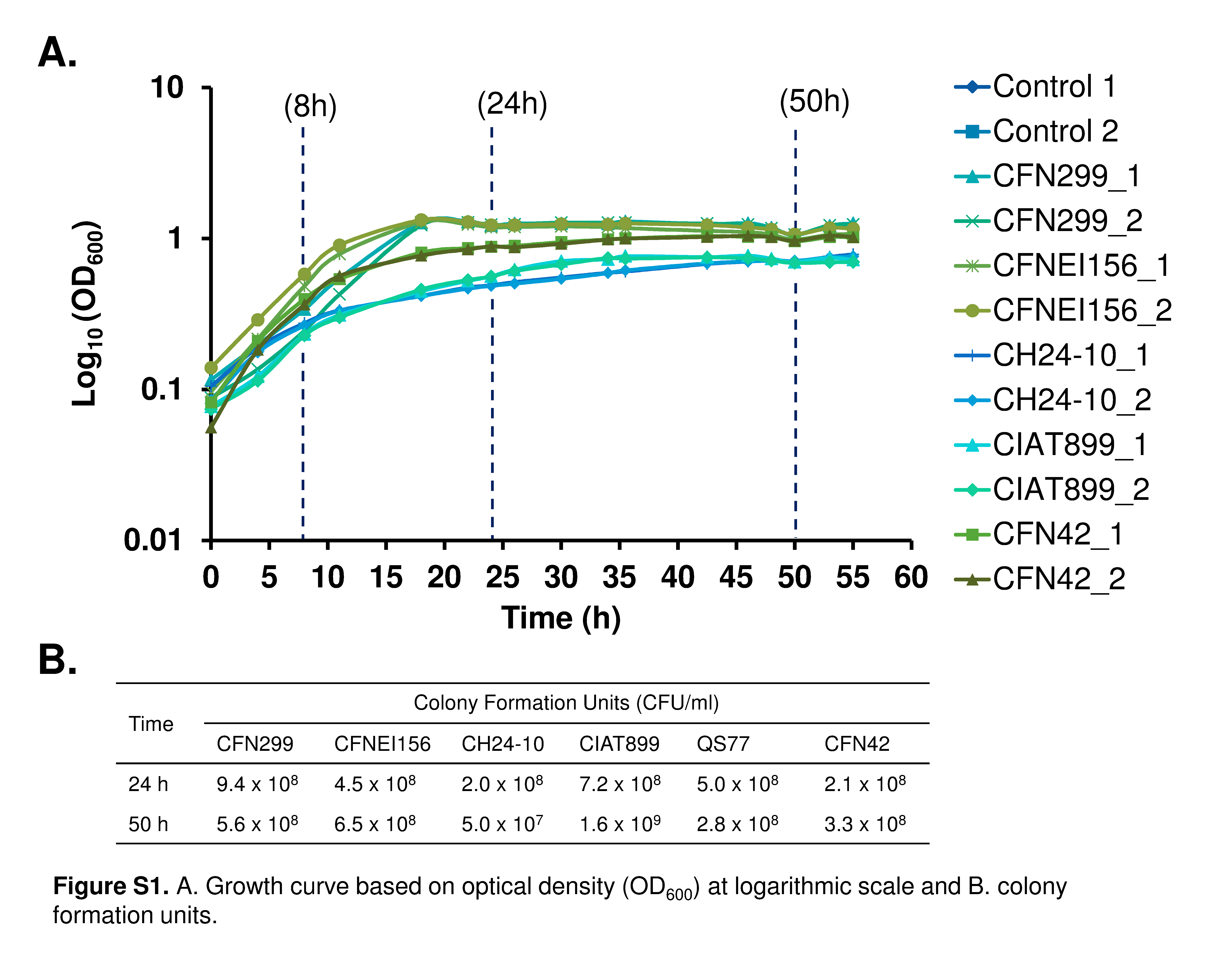

Supplement: Supplementary file 1 [file metabolites-09-00105-s001.zip › FigureS1.tif]
